# Ex situ cultivation protocol for *Cystoseira amentacea* var. *stricta* (Fucales, Phaeophyceae) from a restoration perspective

**DOI:** 10.1371/journal.pone.0193011

**Published:** 2018-02-15

**Authors:** Annalisa Falace, Sara Kaleb, Gina De La Fuente, Valentina Asnaghi, Mariachiara Chiantore

**Affiliations:** 1 Department of Life Sciences, University of Trieste, Trieste, Italy; 2 Department of Earth, Environment and Life Sciences, University of Genova, Genova, Italy; University of Barcelona, SPAIN

## Abstract

Due to multiple impacts, *Cystoseira* forests are experiencing a significant decline, which is affecting the ecosystem services they provide. Despite conservation efforts, there is an urgent need to develop best practices and large-scale restoration strategies. To implement restoration actions, we developed an *ex situ* protocol for the cultivation of *Cystoseira*. *amentacea* var. *stricta*, aimed at reducing the time needed for laboratory culture, thus avoiding prolonged maintenance and minimizing costs. Specifically, we tested the effects of temperature, light and substratum on settlement and growth of early life stages using a factorial experiment. Temperature (20 and 24°C) and photoperiod (15L:9D) were selected to reflect the conditions experienced in the field during the reproductive period. Two light intensities (125 and 250 μmol photons m^−2^s^−1^) were selected to mimic the condition experienced in the absence of canopy (i.e. barren—higher light intensity) or in the understory (lower light intensity) during gamete release. The tested substrata were flat polished pebbles and rough clay tiles. The release of gametes and the successive survival and development of embryo and germlings were followed for two weeks. Regardless of the culture conditions, rougher tiles showed higher zygote settlement, but the substrata did not affect the successive development. *Z*ygote mortality after one week averaged 50% and at the end of the second week, embryonic survival was higher under lower light and temperature conditions, which also determined the growth of larger embryos.

## Introduction

The genus *Cystoseira* C. Agardh, brown algae belonging to the order Fucales, is distributed along the Mediterranean and Atlantic coasts from the intertidal to the lower sublittoral. This genus is ecologically relevant as an “ecosystem engineer” [1], and plays a key functional role in controlling spatial habitat heterogeneity, productivity, and nutrient cycling in temperate rocky reefs. In particular, *Cystoseira* forests provide refuge and food for many invertebrates and fishes and modulate the structure of the associated benthic community [2]. Currently, some *Cystoseira* populations (depending on species and location) are declining/lost throughout the Mediterranean [3,4,5,6,7, 8, 9], largely due to multiple human impacts such as urbanization, overfishing and climate change. Consequently, many systems have shifted from complex and productive assemblages to simpler, less-productive habitats such as barrens, turf-forming algae and other ephemeral opportunistic seaweeds, thus impacting the provision of ecosystem services [3,10–16]. *Cystoseira* species are listed as “of community interest” according to the Habitat Directive (92/43/EEC) [17], and are indicators of environmental quality in Mediterranean coastal waters according to the Water Framework Directive (2000/60/EC) [18] (i.e., EEI [19] and CARLIT [20, 21]. Several species are protected by the Bern Convention, recognized as a priority by the Barcelona Convention and considered vulnerable by several international organizations (i.e. IUCN, RAC/SPA, MedPan).

Despite the implementation of significant conservation efforts, most degraded systems have not recovered, emphasizing the urgency to develop an active intervention to restore endangered habitats [16]. The threat of losing *Cystoseira* species is magnified by their low dispersal capacity due to rapid egg fertilization and zygote sinking [22–25], which hampers natural recovery in the absence of adults, even if in some *Cystoseira* species the potential dispersal distance can be enhanced by the transport in floating rafts [26,27]. As a result, interest in habitat restoration is increasing according to the Biodiversity Strategy to 2020 (Target 2; European Commission, 2011), which recommends the reintroduction of relevant species into areas where they were present historically and where the factors that led to their loss have been removed.

Small-scale *Cystoseira* transplants have been attempted utilizing several techniques [28–31]. The most frequently tested method is the transplantation of juveniles or adult thalli: the only major challenge to this approach is the appropriate fixing of individuals or installation in the target area.

Outplanting, which consists of producing recruits from fertile material in hatcheries for placement in the sea, has been explored for the genus *Cystoseira* to a lesser degree [29,32]. In contrast, many studies have been performed using other large fucoid seaweeds [33–41] with a particular focus on the long-term maintenance of seedlings in culture [41–47].

Usually, the need for large numbers of germlings for outplanting represents a bottleneck in the design of large-scale restoration actions, so it is especially challenging to plan an efficient, effortless and cost-effective seedling production system that fits the breeding needs of a specific species. Considering the high potential of *Cystoseira* to generate gametes and zygotes under optimal conditions, the cultivation of germlings starting from fertile receptacles represents a sustainable option for restoring endangered species without depleting natural populations. From this perspective, the development of effective cultivation protocols tailored to the eco-physiological needs of different species is a compulsory milestone.

The aim of this study was to develop an *ex situ* protocol for the restoration of *Cystoseira amentacea* var. *stricta* Montagne, a sensitive caespitose intertidal Mediterranean species whose reduction/loss has primarily been recorded in several locations in the NW Mediterranean [6,48]. The protocol aimed to maximize zygote settlement, minimize embryo development time and generate a dense coverage of healthy germlings for outplanting. Based on these objectives, we tested the effects of easily adjustable variables (temperature, light and substratum) on the settlement and growth of early life stages to develop best practices for the restoration of this sensitive species.

## Materials and methods

**Fig 1:**
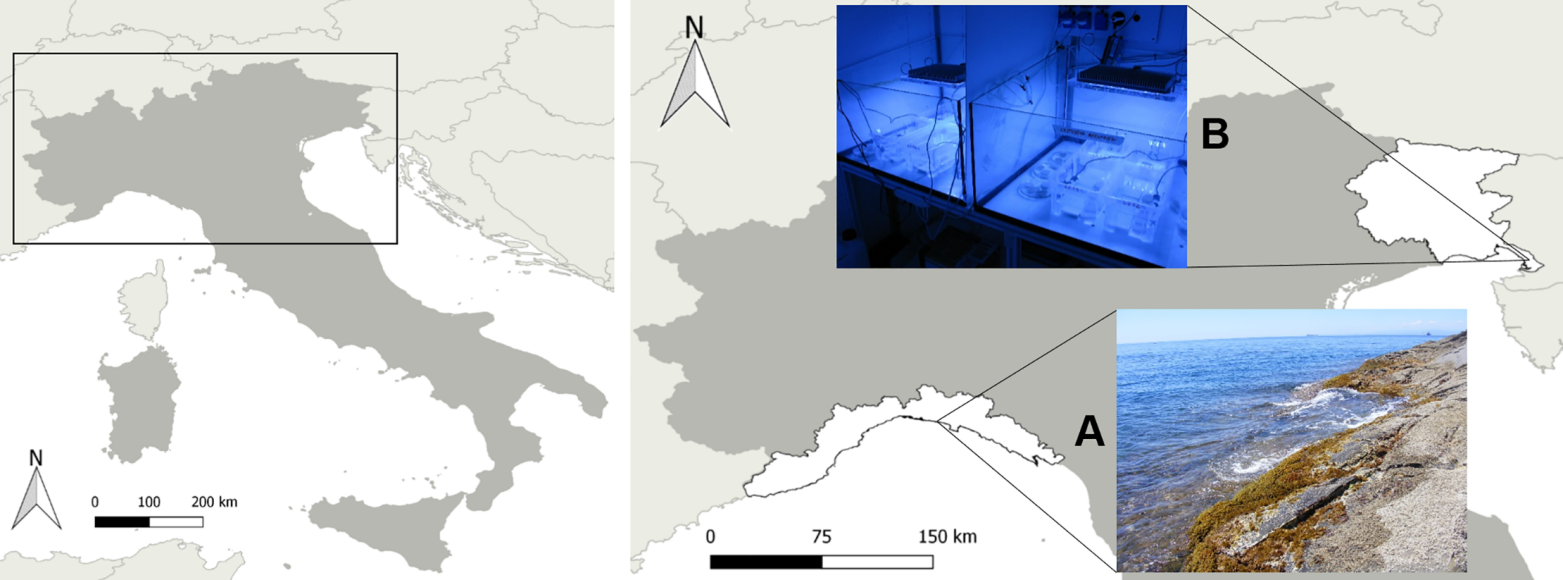
Map showing the geographical location of the collection site (A) and the laboratory site (B) in northern Italy.

In June 2016, during the reproductive period of *C*. *amentacea* var. *stricta*, healthy apical fronds of ca. 3 cm in length holding mature receptacles were collected in the intertidal zone at Bogliasco, Genoa (NW Italy, 44°22'40.37"N—9°4'35.14"E) (Fig 1). No specific permits were required for collecting specimens in the selected location because it is not part of a protected or private area. Additionally, non-destructive sampling was performed, as only apical branches were collected. In particular, the site is characterized by a tide in the range of 30 cm (the barometric tide may dominate the water level) and an average spring temperature of 20°C. After sampling, apices wrapped with seawater-wetted towels were delivered within 24 h under dark, cold and humid conditions to the laboratory in Trieste (NE Italy) (Fig 1) for culture in environmentally controlled rooms.

The temperature and photoperiod were selected to reflect typical seasonal conditions at the sampling site during the reproductive phase of *C*. *amentacea* var. *stricta* (from late spring to summer). The photoperiod was set to a 15:9 h light:dark cycle, and light intensities were chosen to mimic two possible scenarios occurring in nature during the gamete release, fertilization and the early life growth stages of *Cystoseira*: in the absence of a canopy, as on barren ground (higher light intensity) or in the understory (lower light intensity). Light was provided by LED lamps (AM366 Sicce USA Inc., Knoxville, USA), and irradiance was measured with a LI-COR LI-190/R Photometer (LICOR-Biosciences, Lincoln, NE, USA). Light irradiance (L) was set at 125 μmol photons m^−2^s^−1^ (L^-^) or at 250 μmol photons m^−2^s^−1^ (L^+^), while temperature (T) was set at 20°C (T^-^) or at 24°C (T^+^) The medium used in the experiments was Stosch's enriched filtered and autoclaved seawater (VSE) [49,50]. Aquaria were filled with 4 L of culture medium, renewed every 4 days to minimize any possible effects of nutrient limitation and continuously aerated by air pumps. Two substrata with differing natures and roughness were tested: flat polished pebbles and rough clay tiles.

A factorial laboratory experiment was performed that combined irradiance, temperature and substratum. Four combinations of culturing conditions consisting of two crossed levels of each environmental condition (L^+^T^+^, L^+^T^-^, L^-^T^+^, L^-^T^-^) and two substrata (Pebbles and Tiles) were tested in a two-way crossed design.

Fertile apices were gently cleaned with a brush and rinsed with sterile seawater to remove adherent biofouling and surface detritus. Fronds were then placed in individual aquaria: three apices with mature receptacles on each substratum in separate aquaria per condition (in triplicate). Three additional replicates were placed on glass slides under each condition to observe zygote development with an inverted microscope (Leica, DM IL LED), and photographs were obtained with a Canon Powershot G9, avoiding stress on the treatment replicates.

### Data analysis

#### Egg release and settlement

After 2 h, gametes were released on all substrata under all conditions. Next, the receptacles were removed, and their fresh weight (FW) was measured. Due to the high density of eggs released on each substratum and to reduce manipulation stress as much as possible, counts were performed by processing photographic data. For each substratum, eggs were counted in 5 randomly selected 1x1 cm quadrants in photos obtained from a Leica MZ6 stereo microscope with a Nikon Coolpix 4500 camera. The number of eggs per unit of receptacle FW was analyzed as a response variable to compare settlement on different substrata under different conditions. Two-way crossed ANOVA was performed using both factors and their interaction as fixed factors. The data were square-root transformed to satisfy the assumptions of normality and homoscedasticity.

#### Embryo development

**Fig 2:**
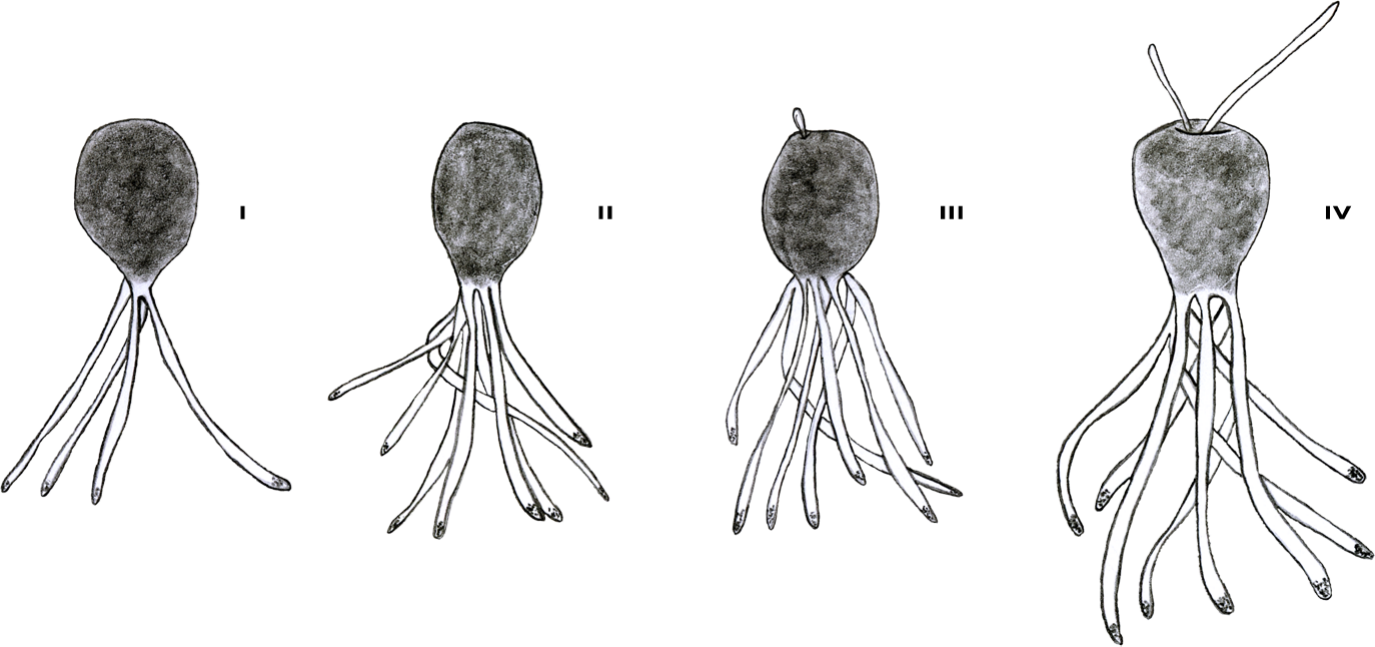
Early stages of *C*. *amentacea* var. *stricta*at week 1: I-embryos with 4 primary rhizoids, II-embryos with 8 rhizoids, III-embryos with short apical hair/s, and IV-embryos with long apical hair/s.

At the end of the first week, replicate embryos in all four growth stages were visible on glass slides and were counted: I-embryos with 4 primary rhizoids, II-embryos with 8 rhizoids, III-embryos with short apical hair/s, and IV-embryos with long apical hair/s (Fig 2). To analyze the differences among conditions, a PERMANOVA was applied using the percentage of individuals at each stage as a response variable and conditions as factors. Pairwise comparisons of significant terms were performed.

#### Embryo survival

After 24 h (T0), the number of zygotes was counted on each substratum (5 1x1 cm quadrants) by processing photographic data. Counts were then repeated at week 1 and week 2 to calculate the germling survival rate. We applied an ANCOVA for both week 1 and week 2 with unequal slopes for survival rate as a response variable and density (i.e., number of fertilized eggs) as a covariate, and substrata, conditions and their interactions were used as fixed factors. Assumptions were validated after applying the arcsine square root transformation (suitable for proportional data). Post hoc SNK tests were performed on significant interaction terms.

#### Subsequent germling growth

**Fig 3:**
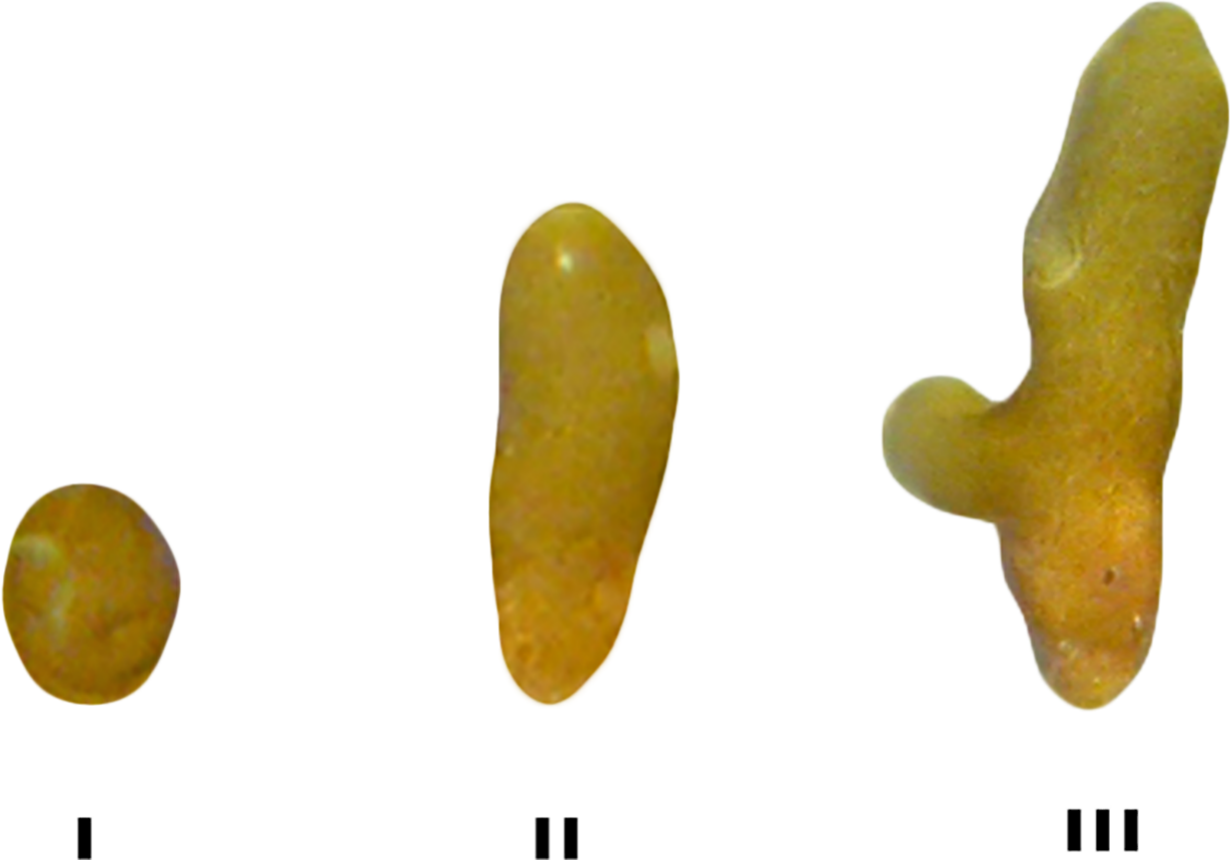
*C*. *amentacea* var. *stricta* germling stages at week 2: I-round-shaped, II-elongated, and III- elongated with branching.

At week 2, three subsequent developmental stages were identifiable: I-round-shaped, II-elongated, and III-elongated with branch (Fig 3). The area of ten randomly chosen individuals per shape was measured in each replicate substratum and used as a response variable. The area was quantified by processing photographic data using ImageJ software [51]. Conditions and substrata were used as crossed fixed factors in a PERMANOVA, and pairwise comparisons were performed on significant terms.

## Results

### Morphogenesis

**Fig 4:**
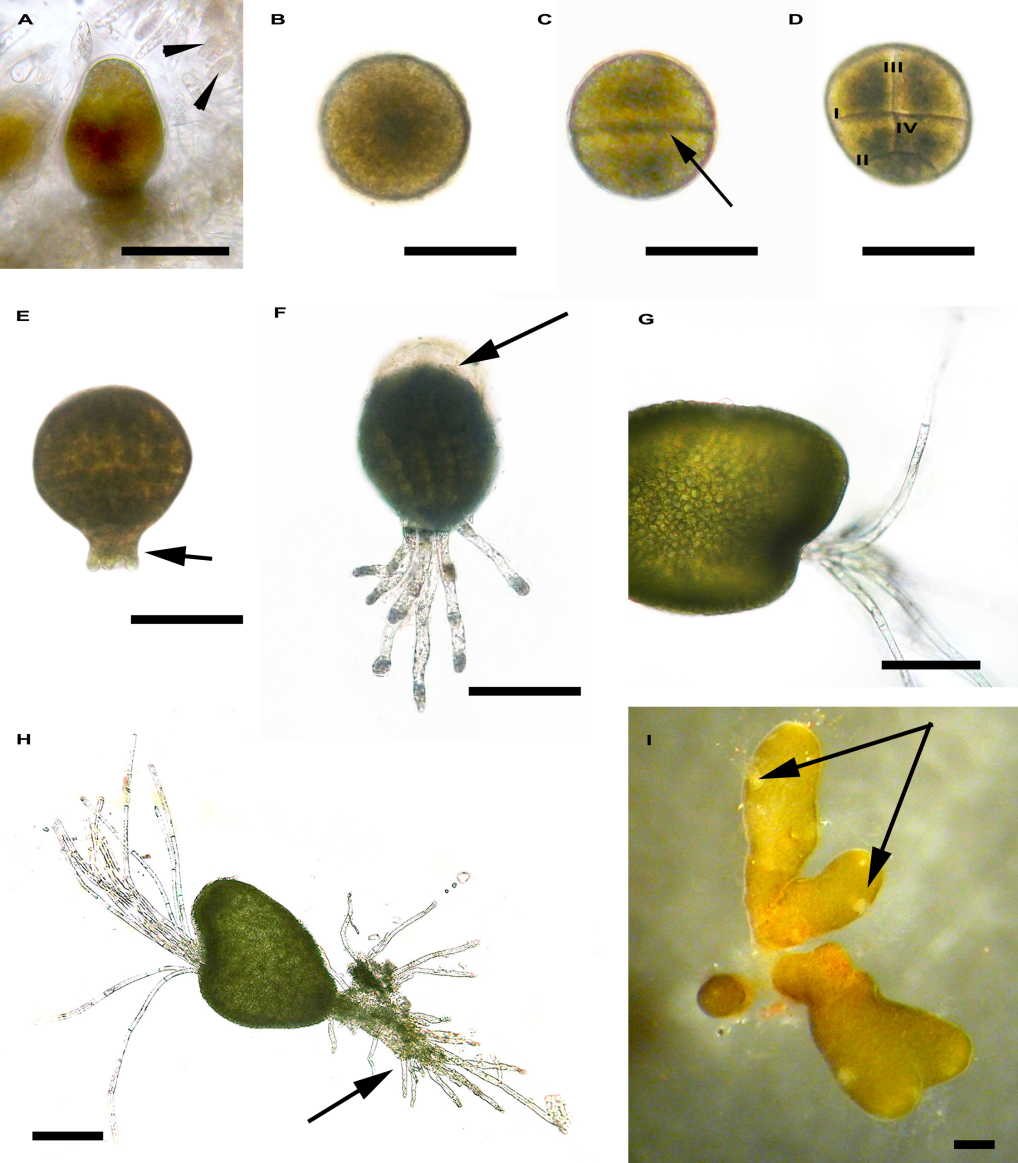
Early development of *Cystoseira amentacea* var. *stricta*. A. Detail of a conceptacle with an oogonium and antheridia (arrowhead). B. Zygote with a central large nucleus. C. First zygote division (arrow). D. Second zygote division (II) parallel to the first (I) and third (III) and fourth divisions (IV) perpendicular to the first. E. Embryo with rhizoidal buds (arrow). F. Embryo with secondary rhizoids. Note the detachment of the fecundation membrane (arrow) during embryo elongation. G. Hyaline hairs growing from the invagination in the apical region of the embryo. H. Embryo with long apical hairs and numerous rhizoids (arrow). I. Germling with cryptostomata (arrows). Bar = 200 μm.

*C*. *amentacea* var. *stricta* is a monoic species with female and male gametes produced in the same conceptacle (Fig 4A). In our trials, gamete release began soon after the receptacles were placed in the aquaria, and the mean diameter of the eggs was 122±3 μm (n = 20). Fertilization occurred externally, and the development of a fecundation membrane around the zygote facilitated its adhesion to a substratum (Fig 4B). The zygote cytoplasm, which was initially homogeneous, became metabolically differentiated (polarization) with the establishment of a vertical growth axis (connecting the rhizoid and thallus pole). Twelve hours after fertilization (AF), the first division perpendicular to the growth axis was observed, leading to the formation of two equally sized cells (Fig 4C). The second division, which was parallel to the first, occurred in the lower cell 20–22 h AF (Fig 4D), while the third division, perpendicular to the first, appeared in the upper cell (Fig 4D). Within 32–34 h AF, many divisions occurred without an increase in embryo volume.

Within the first week, the rhizoids developed as follows: via perpendicular divisions, the rhizoid mother cell gave rise to four cells that differentiated into four primary rhizoids (Figs 4E and 2) that grew further, forming long filaments (ca. 150–200 μm long). After detachment of the fecundation membrane, the length of the embryo increased through subsequent cell divisions, and secondary rhizoids were formed (Fig 4F). Thus, the embryo assumed an erect position, and an invagination with hyaline hairs appeared in the apical region (Fig 4H and 4G). At week 1 AF, the more developed embryos were 353±26 μm long and 259±37 μm wide (n = 20). At week 2 AF, germlings with numerous rhizoids grew further [466±26 μm long and 275±28 μm wide (n = 20)] and small lateral branches with some cryptostomata began to appear. At week 3 AF, numerous cryptostomata were observed (Fig 4I), and iridescence, which is typical of adult plants, was visible on the thallus surface. At this time point, the germlings were 1.38±0.13 mm long and 0.46±0.06 mm wide (n = 20). At the end of the third week, few tiles were transported back in the field at Bogliasco. Juveniles were 4.73±0.05 mm long and 0.81±0.09 mm wide after 1 month in the field, and they grew up to 9 cm in 9 months (April 2017).

### Egg release and settlement

The number of settled eggs was higher on Tiles (avg = 5226, SE = 566) than on Pebbles (avg = 2429; SE = 199), highlighting a significant effect of substratum roughness (p<0.0001; S1 Table). Conversely, no significant differences were detected between conditions or within the interaction term.

### Early embryo development

**Fig 5:**
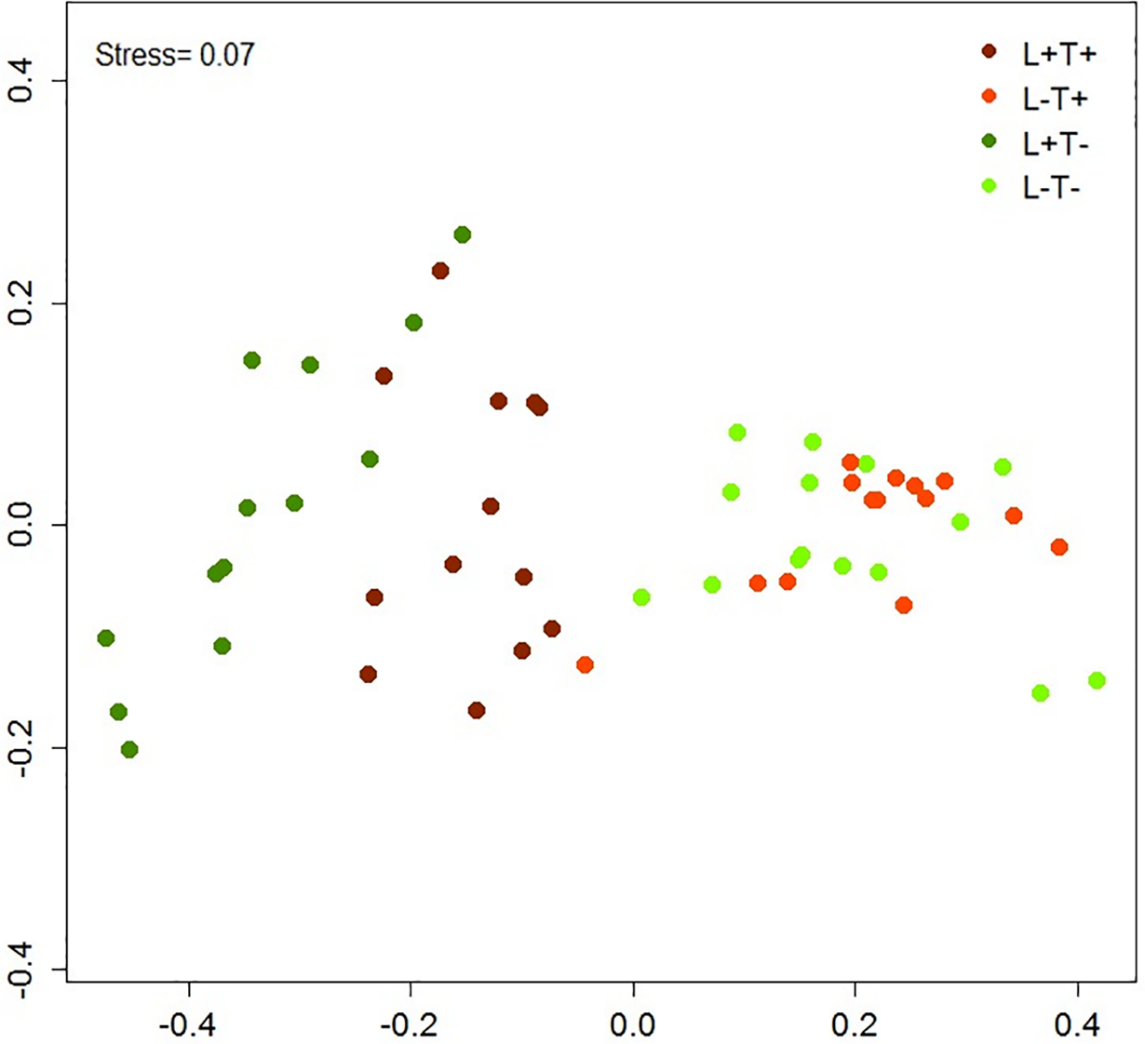
MDS ordination plot of the percent composition of embryonic developmental stages at week 1 under the different conditions. L^+^ T^+^ = high light–high temperature, L^+^ T^-^ = high light–low temperature, L^-^ T^+^ = low light–high temperature, L^-^ T^-^ = low light–low temperature.

**Fig 6:**
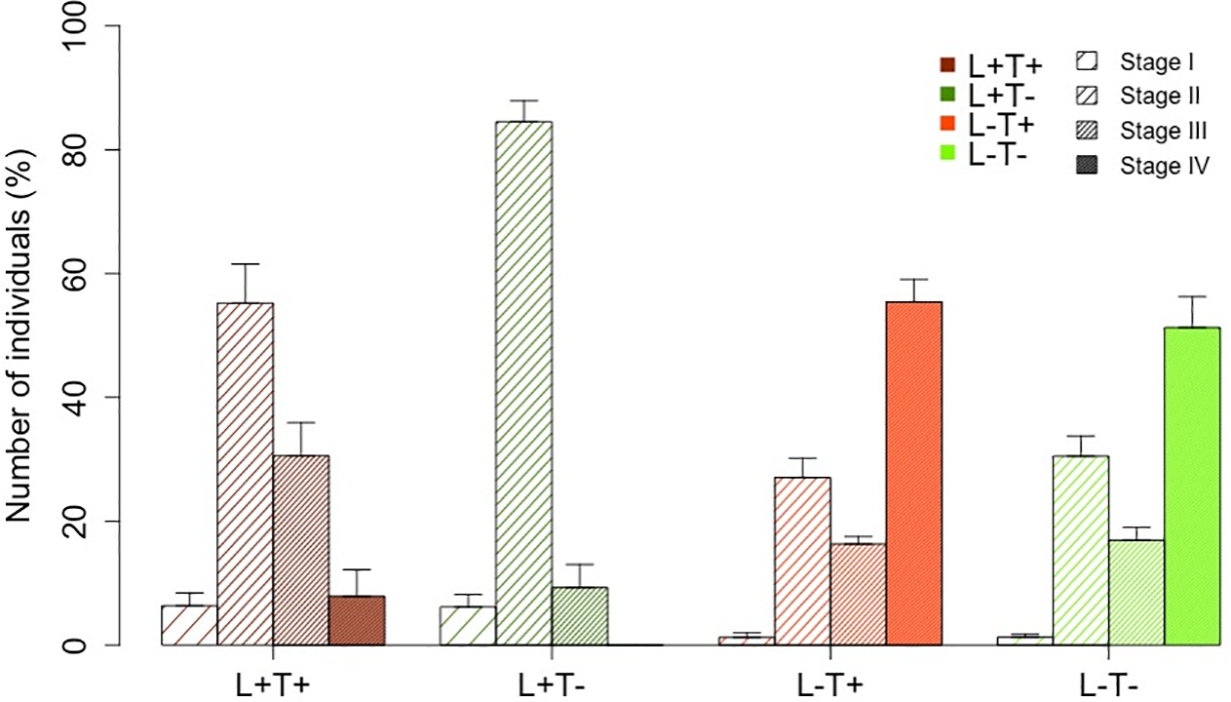
Bar plot of the percent composition of embryonic developmental stages at week 1 under the different conditions. L^+^ T^+^ = high light–high temperature, L^+^ T^-^ = high light–low temperature, L^-^ T^+^ = low light–high temperature, L^-^ T^-^ = low light–low temperature. I = embryos with 4 primary rhizoids, II = embryos with 8 rhizoids, III = embryos with short apical hair/s, IV =: mbryos with long apical hair/s.

The percentage of individuals was calculated for each of the four stages observed in the glass slide replicates (Fig 2). An MDS ordination plot (Fig 5) showed three different groups: L^+^T^-^, L^+^T^+^ and one group comprising L^-^ conditions (L^-^T^-^ and L^-^T^+^). PERMANOVA confirmed significant differences among these groups (p<0.001; S2 Table). Furthermore, a bar plot (Fig 6) revealed a higher percentage of embryos in stage IV (embryos with long apical hair/s) under L^-^conditions.

### Embryo survival

**Fig 7:**
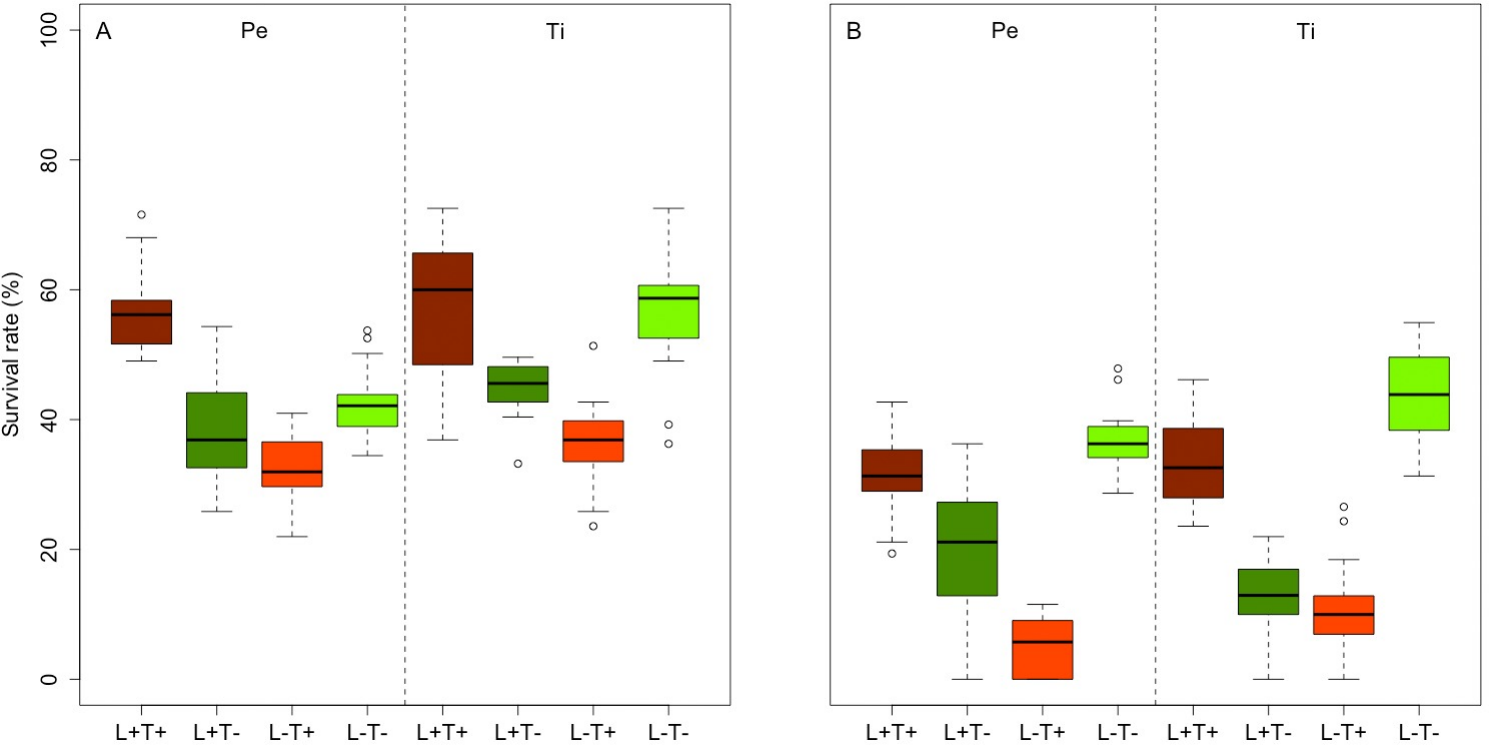
**Boxplot of the survival rates at week 1 (A) and week 2 (B) among conditions and substrata.** L^+^ T^+^ = high light–high temperature, L^+^ T^-^ = high light–low temperature, L^-^ T^+^ = low light–high temperature, L^-^ T^-^ = low light–low temperature. Pe = Pebbles, Ti = Tiles.

The ANCOVA results indicated strong significant differences among conditions and small differences between the two substrata at week 1 (p<0.0001 and p<0.03, respectively; S3 Table). At week 2 the interaction term (conditionXsubstratum) was significant (p<0.001; S4 Table): on both substrata, the extreme conditions did not differ from each other (L^+^T^+^ = L^-^T^-^), but they differed significantly from the other two conditions (L^+^T^-^; L^-^T^+^). On Tiles, L^+^T^-^ and L^-^T^+^ did not differ significantly, although they differed on Pebbles. Additionally, ANCOVA indicated that L^+^T^-^ was significantly different between substrata, while survival was slightly higher on Pebbles. As shown in boxplots (Fig 7), the survival rate at week 1 was higher under extreme conditions (L^+^T^+^; L^-^T^-^) compared to the other two conditions (L^+^T^-^; L^-^T^+^). The survival rate from week 1 to week 2 conspicuously decreased (between 50 and 95%) for the L^+^T^+^, L^+^T^-^ and L^-^T^+^ conditions, while the survival rate under L^-^T^-^ remained more stable with a mortality below 30%.

### Subsequent germling growth

**Fig 8:**
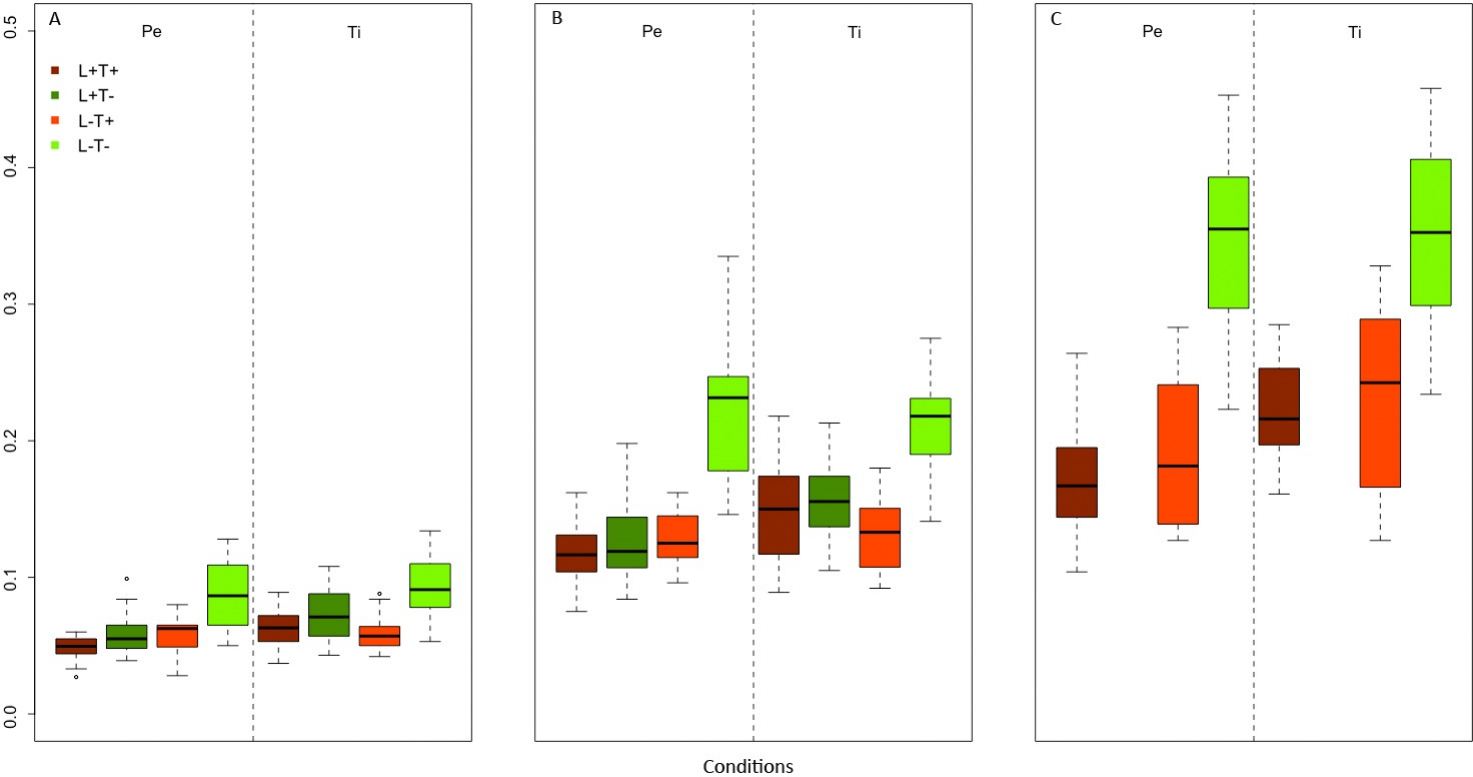
**Boxplots of the area of the different stages (A: stage I; B: stage II; C: stage III) at week 2 among conditions for both substrata.** L^+^ T^+^ = high light–high temperature, L^+^ T^-^ = high light–low temperature, L^-^ T^+^ = low light–high temperature, L^-^ T^-^ = low light–low temperature. Pe = Pebbles, Ti = Tiles.

PERMANOVA performed on the germling area at different stages (Fig 3) at week 2 showed significant differences among conditions (p = 0.001) with L^-^T^-^condition different from the others conditions (S5 Table; Fig 8).

## Discussion

Given the worldwide concern over the loss of key habitat-forming organisms, such as the large brown macroalgae of the order Fucales, and the downstream cascade effects on the services provided by such organisms, there is an urgent need to develop best practices and restoration strategies. Studies that provide sound information on how to best undertake habitat restoration are crucial for managing coastal ecosystems.

Outplanting appears to be an ecologically sustainable approach that consists of two main steps: culturing germlings in the laboratory and transferring them into the field [37,41,45,52]. For *Cystoseira*, outplanting appears to be a feasible management option that can provide many healthy specimens for re-introduction to the environment without impacting natural populations [29,32,53].

In this study, we focused on the first step of the outplanting process: developing an effective protocol for cultivating the early stages of *C*. *amentacea* var. *stricta*. This approach is challenging because most eco-physiological studies of *Cystoseira*, both in the field and in the laboratory, have focused on the adult stages. Nevertheless, the single/few-celled stages are characterized by simplicity and sensitivity, so compared to adults, any environmental variable will exert greater effects on germling mortality and growth rate [54,55]. Thus, the needs of these ontogenetic stages must be understood because findings related to the macrothallus stages cannot be extrapolated to the microscopic stages [56]. Thus, species-specific best practices for the cultivation of germlings must be developed to implement a successful large-scale restoration strategy.

First, we tested whether it was possible to collect samples far from the breeding facility (ca. 600 km) without damaging the reproductive materials to exclude the possible negative effects related to the distance of the target site from the hatchery. Transporting under dark and cold conditions allowed immediate gamete release, thus avoiding thermal and light shock in the laboratory; indeed, receptacles that were placed in aquaria soon after their arrival in the laboratory immediately released gametes. Starting from eggs fecundation, we described the morphogenesis and successive germlings development of *C*. *amentacea* var. *stricta*. Based on zygotes segmentation and number of primary rhizoids, three groups of *Cystoseira* species have been identified by [22]. *C*. *amentacea* var. *stricta* belongs to the first group, that is characterized by spherical eggs, zygotes that adhere to substrata by the fecundation membrane, and four primary rhizoids [57].

Nutrient limitation affects many processes, such as photosynthetic capacity [58], protein content [59,60], photoprotection mechanisms [61–63], egg behavior and settlement, embryonic development and growth rate [64–69], so the culture medium was enriched to allow the germlings to invest their photosynthetic energy in growth processes [70,71]. Better growth of *C*. *amentacea* var. *stricta* germlings with nutrient enrichment has also been observed by [57]. The positive effect of nitrate supply on *C*. *stricta* growth rate has been demonstrated in adults cuttings cultivation, although with small differences between apical and subapical segments [72]. Together with culture conditions, the choice of substrata must also favor the adhesion of gametes and zygotes and their successive development. Regardless of the culture conditions, rougher tiles showed higher zygote settlement than smoother pebbles, although the substrata did not affect successive germling growth or survival under any of the tested conditions.

Embryonic mortality after one week was elevated under all conditions (50% on average), which was expected given the very high stochastic gamete and zygote mortality of *Cystoseira* in the natural environment. At the end of the first week, embryonic survival was positively affected by two of the tested conditions: L^+^T^+^ and L^-^T^-^. At week 2, survival was still higher under the L^-^T^-^ condition but significantly decreased under L^+^T^+^ treatment. The other two combined conditions showed the lowest embryonic survival throughout the entire experiment. Low-light and low-temperature conditions also favor higher embryonic survival rate in *Sargassum vachellianum* Greville cultivations [73]. Lower light intensity also reduced the time required for embryo development, allowing a greater number of individuals to reach developmental stage IV (larger embryos) within the first week regardless of temperature. After two weeks at low irradiance, lower temperature also strongly determined the growth of larger embryos.

Our findings corroborate that environmental conditions (specifically light and temperature) may interact and exert synergistic or antagonistic effects on physiological responses in unpredictable ways that differ according to developmental stage. The distribution of *C*. *amentacea* var. *stricta* is restricted to the intertidal, so this species is naturally exposed to high levels of irradiance that potentially exceed its light energy requirements, as has been reported for other species that live close to the water surface [70,74–76]. Generally, sun-adapted species [*sensu* 77] develop efficient photoprotection mechanisms to tolerate light stress in addition to dynamic photoinhibition [75,76,78–86].Our study highlighted the light-shade adaptation of *C*. *amentacea* var. *stricta* germlings, which showed enhanced growth at lower irradiance (125 μmol photons m^−2^ s^−1^). Other *Cystoseira* species have been cultivated under different conditions that have primarily depended on laboratory facilities such as *Cystoseira susanensis* Nizamuddin (16±1°C | 40 μmol photons m^−2^ s^−1^ [87] and *C*. *barbata* (Stackhouse) C. Agardh (16–17°C | 120 μmol photons m^−2^ s^−1^ [29]. The morphological development of *C*. *amentacea* var. *stricta* embryos cultivated at 18±1°C and an average light intensity of 70 μmol photons m^−2^s^−1^ has been described by [57]. After ca. 2 months in these conditions, embryos cultivated in seawater were 332.60 ± 22.3μm long, while in VSE medium they were 5.14 ± 0.08 mm [57]. In our experiment germlings cultivated in VSE were 1.38±0.13 mm long after three weeks and 4.73±0.05 mm long after one month in the field. We observed cryptostomata and lateral branches after two weeks of cultivation, while in [57] study cryptostomata were observed after 52 days and ramifications appeared after 106 days.

Studies examining adult thalli of *Cystoseira* have demonstrated the absence of photosynthetic inhibition, even with very high irradiance [88]. In *C*. *barbata* (Stackhouse) C. Agardh f. *aurantia* (Kuetzing) Giaccone, photoinhibition only occurs at irradiances higher than 1500 μmol photons m^−2^s^−1^ [89], while photosynthesis in *C*. *mediterranea* Sauvageau is not saturated at an irradiance of 1600 μmol photons m^−2^s^−1^[75]. Notably, the light requirements of adults should not be extrapolated to the microscopic stages because the presence of non-photosynthetic tissues in complex thalli increases the need for light energy [76], and the irradiance reaching the embryos is restricted by adult fronds in nature [35,42,90]. *Cystoseira* zygotes and germlings settle under adult plants, where they find a protective screen against high irradiance and other stressors. In nature, such community self-protection could be particularly important during spring-summer, when *C*. *amentacea* var. *stricta* produces new recruits in the study area. Conversely, the lower irradiance requirements of germlings permit high-density cultures because self-shading is not a restricting factor.

At higher temperatures (24°C), the proliferation of biofouling was enhanced, particularly at lower light intensity, progressively affecting the development of *C*. *amentacea* var. *stricta* embryos. Based on these results, we determined that the lower values tested for irradiance (125 μmol photons m−2 s−1) and temperature (20°C) were the best hatchery conditions to accelerate the development of high numbers of healthy, large embryos.

Further studies are required to improve the second step in the outplanting process to increase the number of juveniles that can reach the adult stage once they are reintroduced into the field. Grazing pressure, timing and density dependent effects need to be considered to achieve the best restoration results.

## Supporting information

S1 Table Two-way crossed ANOVA performed on the number of eggs per gram. Significant effects are in bold.
(PDF)

S2 Table One-way PERMANOVA performed at week 1 based on the percent composition of three embryonic developmental stages under the different conditions. Significant effects are in bold.
^a^Pairwise comparisons among conditions: L^+^T^-^≠L^+^T^+^≠ L^-^T^-^ = L^-^T^+^.
(PDF)

S3 Table ANCOVA performed at week 1 using survival, substratum and condition as factors and density as a covariate. Significant effects are in bold.
^a^SNK test among conditions: L^+^T^+^≠L^-^T^-^≠L^+^T^-^≠L^-^T.
(PDF)

S4 Table ANCOVA performed at week 2 using survival as a response variable, substratum and condition as factors and density as a covariate. Significant effects are in bold.
^a^SNK test among substrata within condition: Cond. L+T-, T≠S; all other Cond., T = S.
^b^SNK test among conditions within substratum: Sub. S, (L+T+ = L-T-)≠L+T-≠L-T+; Sub. T, (L+T+ = L-T-)≠(L+T- = L-T+).
(PDF)

S5 Table Two-way PERMANOVA performed on the germling area at different stages at week 2. **Condition and substratum are crossed fixed factors.** Significant effects are in bold.
^a^Pairwise comparisons among conditions: L^-^T^-^≠L^+^T^+^ = L^-^T^+^ = L^+^T^-^.
(PDF)
