# Supplementary material for: Ex situ cultivation protocol for Cystoseira amentacea var. stricta (Fucales, Phaeophyceae) from a restoration perspective
Source: PLoS One. 2018 Feb 15;13(2):e0193011. doi: 10.1371/journal.pone.0193011 (PMC5813978; doi:10.1371/journal.pone.0193011)
Supplement: S1 Table — Significant effects are in bold. (PDF) [file pone.0193011.s001.pdf]

	df	SS	MS	F	P
Substratum (S)	1	3020.75	3020.75	33.31	**2.87e-05**
Condition (C)	3	334.16	111.39	1.23	0.33
S:C	3	511.07	170.36	1.88	0.17
Residual	16	1451.29	90.71		
