# Supplementary material for: Ex situ cultivation protocol for Cystoseira amentacea var. stricta (Fucales, Phaeophyceae) from a restoration perspective
Source: PLoS One. 2018 Feb 15;13(2):e0193011. doi: 10.1371/journal.pone.0193011 (PMC5813978; doi:10.1371/journal.pone.0193011)
Supplement: S2 Table — Significant effects are in bold. aPairwise comparisons among conditions: L+T-≠L+T+≠ L-T- = L-T+. (PDF) [file pone.0193011.s002.pdf]

	df	SS	MS	F	R^2	P
Condition	3	2.80	0.93	34.23	0.65	**0.001**[a]
Residual	56	1.52	0.03		0.35	
Total	59	4.32			1.00	
