# Supplementary material for: Ex situ cultivation protocol for Cystoseira amentacea var. stricta (Fucales, Phaeophyceae) from a restoration perspective
Source: PLoS One. 2018 Feb 15;13(2):e0193011. doi: 10.1371/journal.pone.0193011 (PMC5813978; doi:10.1371/journal.pone.0193011)
Supplement: S3 Table — Significant effects are in bold. aSNK test among conditions: L+T+≠L-T-≠L+T-≠L-T. (PDF) [file pone.0193011.s003.pdf]

Week 1	df	SS	MS	F	P
Density	1	1702.2	1702.17	29.62	**$3.18e^{-07}$**
Substratum (S)	1	274.9	274.91	4.78	**0.03**
Condition (C)	3	8404.3	2801.43	48.74	**$2.2e^{-16}$**[a]
S:C	3	367.0	122.34	2.13	0.10
Residuals	111	6379.9	57.48		
