# Supplementary material for: Ex situ cultivation protocol for Cystoseira amentacea var. stricta (Fucales, Phaeophyceae) from a restoration perspective
Source: PLoS One. 2018 Feb 15;13(2):e0193011. doi: 10.1371/journal.pone.0193011 (PMC5813978; doi:10.1371/journal.pone.0193011)
Supplement: S4 Table — Significant effects are in bold. aSNK test among substrata within condition: Cond. L+T-, T≠S; all other Cond., T = S. bSNK test among conditions within substratum: Sub. S, (L+T+ = L-T-)≠L+T-≠L-T+; Sub. T, (L+T+ = L-T-)≠(L+T- = L-T+). (PDF) [file pone.0193011.s004.pdf]

Week 2	df	SS	MS	F	P
Density	1	2321.5	2321.5	48.11	**$2.83e^{-10}$**
Substratum (S)	1	192.7	192.7	3.99	0.05
Condition (C)	3	17125.8	5708.6	118.29	$2.2e^{-16}$
S:C	3	898.5	299.5	6.21	**0.0006**[ab]
Residuals	111	5356.8	48.3		
