# Supplementary material for: Ex situ cultivation protocol for Cystoseira amentacea var. stricta (Fucales, Phaeophyceae) from a restoration perspective
Source: PLoS One. 2018 Feb 15;13(2):e0193011. doi: 10.1371/journal.pone.0193011 (PMC5813978; doi:10.1371/journal.pone.0193011)
Supplement: S5 Table — Condition and substratum are crossed fixed factors. Significant effects are in bold. aPairwise comparisons among conditions: L-T-≠L+T+ = L-T+ = L+T-. (PDF) [file pone.0193011.s005.pdf]

	df	SS	MS	F	R^2	P
Substratum (S)	1	0.01	0.002	0.51	0.01	0.47
Condition (C)	3	0.16	0.05	12.30	0.71	**0.001**[a]
S:C	2	0.01	0.004	0.98	0.04	0.40
Residual	13	0.06	0.004		0.25	
Total	19	0.22			1.00	
